# Effect of Recombinant Cytokines on the Expression of Natural Killer Cell Receptors from Patients with TB or/and HIV Infection

**DOI:** 10.1371/journal.pone.0037448

**Published:** 2012-06-08

**Authors:** Venkata Ramana Rao Parasa, Rajasekaran Sikhamani, Alamelu Raja

**Affiliations:** 1 Department of Immunology, National Institute for Research in Tuberculosis (ICMR), Formerly Tuberculosis Research Centre, Chetput, Chennai, India; 2 Government Hospital of Thoracic Medicine, Tambaram Sanatorium, Chennai, India; New York University, United States of America

## Abstract

**Background:**

NK cells express several specialized receptors through which they recognize and discriminate virally-infected/tumor cells efficiently from healthy cells and kill them. This ability to lyse is regulated by an array of inhibitory or activating receptors. The present study investigated the frequency of various NK receptors expressed by NK cell subsets from HIV-infected TB patients. The effect of IL-15+IL-12 stimulation on the expression of NK receptors was also studied.

**Methodology/Principal Findings:**

The study included 15 individuals each from normal healthy subjects, pulmonary tuberculosis patients, HIV-infected individuals and patients with HIV and tuberculosis co-infection. The expression of NK cell receptors was analyzed on two NK cell subsets within the peripheral blood: CD16+CD3− and CD56+CD3− using flow cytometry. The expression of inhibitory receptors (CD158a, CD158b, KIRp70, CD85j and NKG2A) on NK subsets was increased in HIV, when compared to NHS. But the response in HIV-TB was not uniform. Stimulation with IL-15+IL-12 dropped (*p*<0.05) the expression of CD85j and NKG2A in HIV. The basal expression of natural cytotoxicity receptors (NKp30 and NKp46) on NK cell subsets was lowered (*p*<0.05) in HIV and HIV-TB as compared to NHS. However, the expression of NKp44 and NKG2D was elevated in HIV. Enhanced NKp46 and NKG2D expression was observed in HIV with IL-15+IL-12 stimulation. The coreceptor NKp80 was found to be expressed in higher numbers on NK subsets from HIV compared to NHS, which elevated with IL-15+IL-12 stimulation. The expression of NK receptors and response to stimulation was primarily on CD56+CD3− subset.

**Conclusions/Significance:**

IL-15+IL-12 has an immunomodulatory effect on NK cell subsets from HIV-infected individuals *viz* down-regulation of iNKRs, elevation of activatory receptors NKp46 and NKG2D, and induction of coreceptor NKp80. IL-15+IL-12 is not likely to be of value when co-infected with TB probably due to the influence of tuberculosis.

## Introduction

Natural Killer (NK) cells represent a highly specialized lymphoid population that lack antigen specific receptors, but can lyse tumor and virus-infected cells, without prior sensitization [1]. They commonly express a variety of nonexclusive phenotypic markers such as CD16, CD56, CD57, and to some extent CD8 [2]. Unlike T and B lymphocytes, NK cells do not rearrange genes encoding receptors for antigen recognition, but they have developed the ability to recognize self-MHC class I or class I–like molecules through a unique class of receptors, NK cell receptors (NKRs), that can inhibit or activate NK cell killing [3].

The effector functions of NK cells are finely regulated by a series of inhibitory or activating receptors [4]. The inhibitory receptors, specific for major histocompatibility complex (MHC) class I molecules, allow NK cells to discriminate between normal cells and cells that have lost the expression of MHC class I. Accordingly, lack of interaction of these receptors with MHC class I molecules may result in the killing of the target cells [5]. This occurs when target cells have lost or express insufficient amounts of MHC class I molecules. The inhibitory form of NK receptors provides the protective immunity through recognizing class I MHC molecules with self-peptides on healthy host cells. The activating or the noninhibitory NK receptors mediate the killing of tumor or virally infected cells through their specific ligand recognition [6].

The inhibitory NK receptors (iNKRs) which are responsible for delivering inhibitory signal include the human killer cell Ig-like receptors (KIRs), leukocyte Ig-like receptors (LIRs) and members of NKG2 family. All the iNKRs have characteristic immuno tyrosine inhibitory motifs (ITIM) in their intracellular domain through which the intracellular signal transduction takes place. In contrast, activating receptors carry immuno tyrosine activation motifs (ITAM) in their cytoplasmic domain. Activating receptors include 2B4, the natural cytotoxicity receptors- NKp46, NKp30, and NKp44, the noninhibitory isoforms of KIRs and LIRs, CD94/NKG2C and CD94/NKG2E heterodimers, and NKG2D homodimer.

Despite extensive progress in the recent years, many of the intriguing and specific NK receptors are still poorly understood with respect to ligand specificity and signaling properties. NK cells from HIV-infected individuals had been shown to exhibit reduced natural cytotoxicity [7] and more so in patients co-infected with TB [8]. The present study hypothesizes that the NK receptors, which are known to involve in cytotoxicity might be affected during HIV infection. In this context, the expression profile of various NK receptors in infected and un-infected individuals was studied separately on CD56+ and CD16+ cells.

The stimulatory effect of cytokines on NK activity has been well documented [9]. IL-2–activated NK cells are known to increase the lytic activity compared with circulating NK cells and are able to lyse otherwise NK cell–resistant targets [10]. The potential effects of IL-15+ IL-12 on the enhancement of NK activity in HIV positive individuals have been demonstrated by us [8], [11], [12]. Hence, the present study assessed the effect of IL-15+ IL-12 on the expression of NK surface receptors in HIV positive individuals with and without TB co-infection.

## Materials and Methods

### Study Participants and Ethics

This study was approved by the Institutional ethics committee. All the study participants were informed about the study protocol and gave written consent to participate in this study. Patients were recruited from the Government Hospital of Thoracic Medicine, Tambaram Sanatorium, Chennai between September 2007 and March 2009. Healthy volunteers from the laboratory were recruited as normal healthy subjects.

The study participants consist of four groups which included normal healthy subjects [NHS, N = 15, age = 22–29 (median = 23)], pulmonary tuberculosis patients [TB, N = 15, age = 22–55 (median = 39)], HIV-infected individuals [HIV, N = 15, age = 23–44 (median = 30)] and HIV co-infected tuberculosis patients (HIV-TB, N = 15, age = 26–46 (median = 34)]. The TB patients were diagnosed based on clinical manifestation typical for *M. tuberculosis,* radiologic analysis and sputum smear positivity. HIV seropositivity was determined by 2 rapid EIAs: HIV TRI-DOT (J. Mitra & Co, India) and Retroquic (Qualtrodiagnostics, India). CD4 count ranged from 13 to 643 cells/mm^3^ (median 205) for HIV and 12 to 388 cells/mm^3^ (median 141) for HIV-TB. All the subjects recruited in the study were naïve for anti-tuberculous and anti-retroviral therapy.

### Separation of PBMCs

The PBMCs were separated from blood samples by density gradient centrifugation using Histopaque (Sigma-Aldrich Corp, St. Louis, MO, USA). The cells were washed twice with Hanks balanced salt solution (Sigma-Aldrich Corporation, St. Louis, MO, USA) and resuspended at a density of 2×10^6^ cells/ml in RPMI-1640 (Sigma-Aldrich Corporation, St. Louis, MO, USA) supplemented with heat inactivated 10% human AB serum (Sigma-Aldrich Corporation, St. Louis, MO, USA) and 1× antibiotic/antimycotic solution (Invitrogen Corporation, CA, USA).

### *In vitro* Culture of PBMCs with Recombinant Cytokines

PBMCs at a density of 2×10^6^ cells/ml were cultured overnight in 24 well tissue culture plates (Costar, Corning Inc., NY, USA) in the presence and absence of purified rhIL-15 (BD Biosciences, San Jose, CA, USA) and purified rhIL-12 (R&D Systems Inc., MN, USA) at a concentration of 10 ng each per ml of culture at 37°C, in 5% CO_2_ atmosphere. The optimum concentration required for stimulation was determined in preliminary experiments (**data not shown**).

### Measurement of NK Surface Receptors

The cultured cells were washed twice using 1× PBS and incubated with purified mouse anti-human antibodies specific for CD158a, CD158b, CD85j, CD94, NKp80 and NKG2D (BD Biosciences, San Jose, CA, USA); KIRp70 and NKG2A (Immunotech, Fullerton, CA, USA) for 30 min at 4°C followed by incubation with Phycoerythrin labeled goat anti-mouse IgG antibody (Jackson ImmunoResearch Laboratory, West Grove, PA, USA) for 20 min. PBS containing 1% BSA was used throughout the experiment for blocking Fc receptors. After incubation, cells were stained for other surface receptors CD3-FITC (UCHT1), CD16-PECy5 (3G8), CD56-PECy5 (B159) and CD8-APC (RPA-T8) (BD Biosciences, San Jose, CA, USA), washed and finally resuspended in 0.5% paraformaldehyde. For those tubes with all direct labels CD244 (BD Biosciences, San Jose, CA, USA); NKp30, NKp44 and NKp46 (Beckman Coulter Inc., Fullerton, CA, USA), staining was carried out in a single step. The tubes containing cells were vortexed and acquired in BD FACSCalibur flow cytometer (BD Biosciences, San Jose, CA, USA). The data collected from FACSCalibur was analyzed using FlowJo software (Tree Star Inc., San Carlos, CA, version 7.1.1, USA). At least 35,000 events within lymphocyte gate were collected per sample.

### Statistical Analysis

The data were analyzed using the GraphPad Prism version 4.03 for Windows (GraphPad software, San Diego, CA, USA). Data are presented as mean ± standard error mean (SEM) of 15 subjects in each group. Statistical comparisons between NHS and other groups were performed using one-way ANOVA followed by Tukey’s multiple comparison test and the significant values are denoted by *. Statistical analysis between basal and IL-15+ IL-12 stimulation was carried out using Mann-Whitney U test and the significance is showed as #. A *p* value of <0.05 was considered statistically significant.

## Results

### Expression of Inhibitory NK Receptors (iNKRs)

The proportion of various iNKRs- CD158a, CD158b, KIRp70, CD85j and NKG2A expressed by NK cell subsets were measured and are as follows:

#### CD158a, CD158b and KIRp70

**Figure 1:**
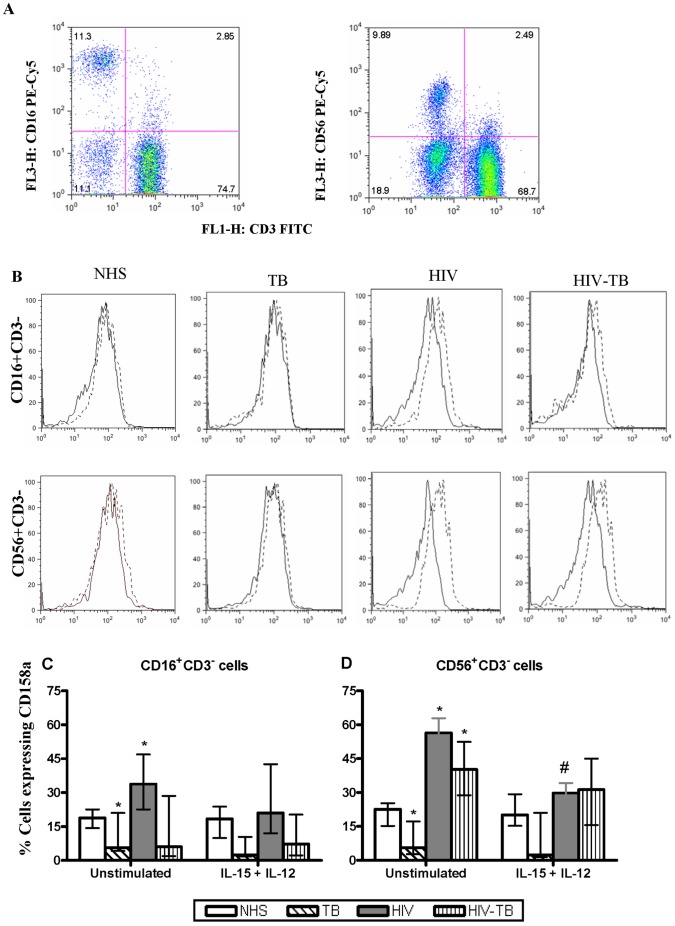
Expression of inhibitory NK receptor CD158a. Dot plots of CD16+ and CD56+ cells (a), representative histograms of CD16+CD3− and CD56+CD3− cells from the four groups showing the expression of CD158a (b). Dashed line in histogram refers to unstimulated while the thick line represents IL-15+IL-12 stimulation. Effect of IL-15+IL-12 on the CD158a expression from four different groups obtained from 15 individuals (c). Data are presented as median ± IQR. Statistical comparisons between NHS and other groups were performed and the significant values are denoted by *. Statistical analysis between basal and IL-15+ IL-12 stimulation were also carried out and the significant values are showed as #. A *p* value of <0.05 was considered statistically significant.

Representative flow cytometric dot plot of CD16+ cells and CD56+ cells (**Fig. 1a**) and the histograms showing the expression of CD158a from NK cell subsets (CD16+CD3− and CD56+CD3−) among four different groups (**Fig. 1b**) are presented. The basal level expression of CD158a was reduced (*p*<0.05) in TB for the NK cells subsets, when compared with NHS. In HIV, the basal CD158a expression was elevated for CD16+CD3− and CD56+CD3− subsets as compared to NHS. As compared to NHS, in HIV-TB patients basal CD158a expression showed a varied response, elevated for CD56+CD3− subset while no significant change for CD16+CD3− cells. After stimulation with IL-15+ IL-12, a generalized reduction of CD158a was observed however significant reduction was found in CD56+CD3− cells from HIV (**Fig. 1c**). The expression pattern of CD158b and KIRp70 on NK cell subsets was similar to CD158a (**data not shown**).

#### CD85j

**Figure 2:**
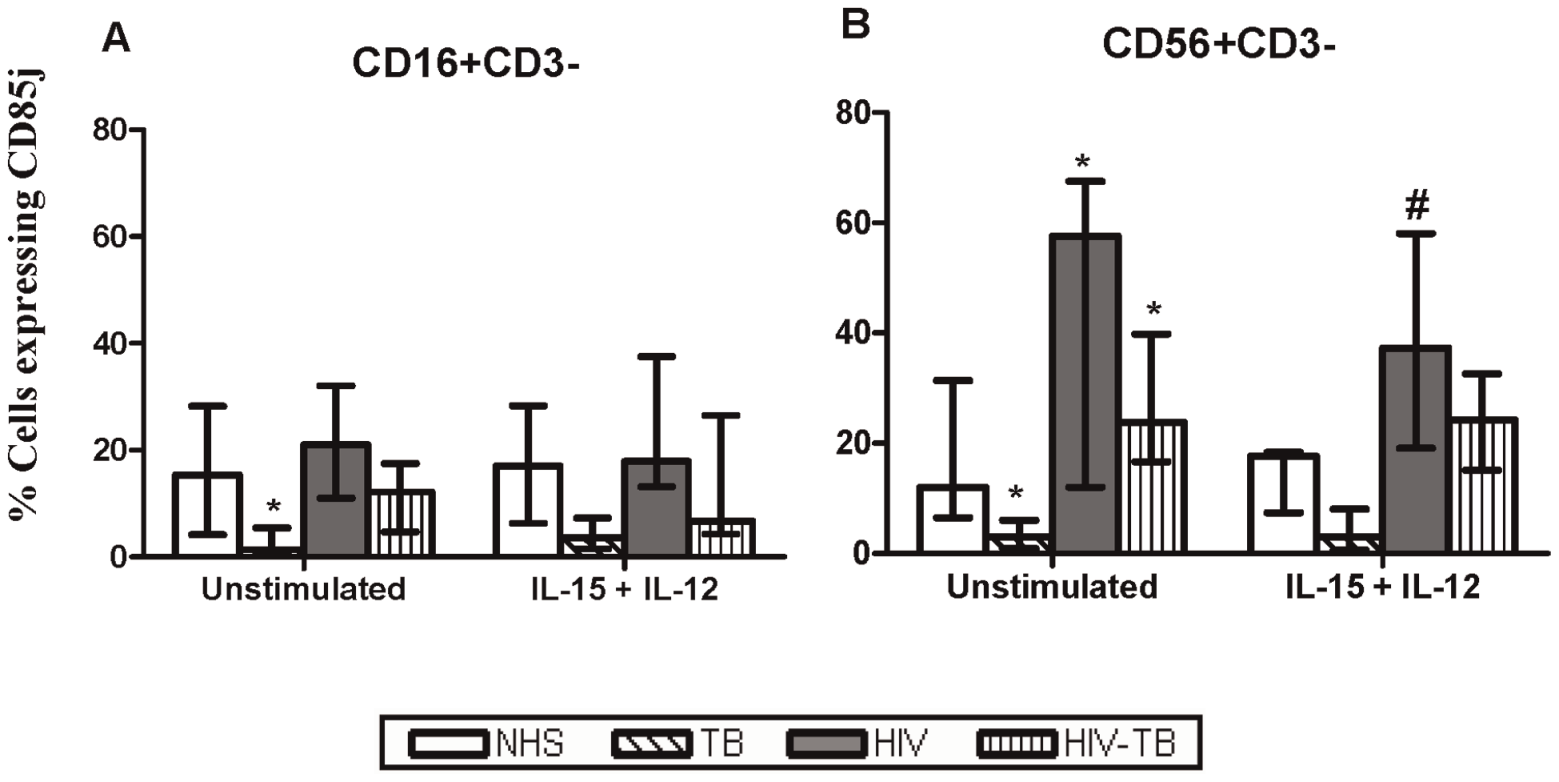
Expression of inhibitory NK receptor CD85j. The CD85j expression on CD16+CD3− and CD56+CD3− cells from different groups in the presence/absence of IL-15+IL-12 stimulation is depicted here. Data are given as median ± IQR from 15 individuals each. Statistical comparisons between NHS and other groups were performed and the significant values (*p*<0.05) are shown as *. Statistical significance (*p*<0.05) between basal and IL-15+ IL-12 stimulation were represented by #.

The TB patients had reduced expression (*p*<0.05) of CD85j on both NK cell subsets compared to NHS. The expression of CD85j in HIV and HIV-TB was found to be elevated (*p*<0.05) on CD56+CD3− cells, while no such changes was observed with CD16+CD3− cells, when compared with NHS. When the cells were treated with IL-15+ IL-12, there was a decline in the expression of CD85j on CD56+CD3− subset in HIV-infected individuals (**Fig. 2**).

#### NKG2A

**Figure 3:**
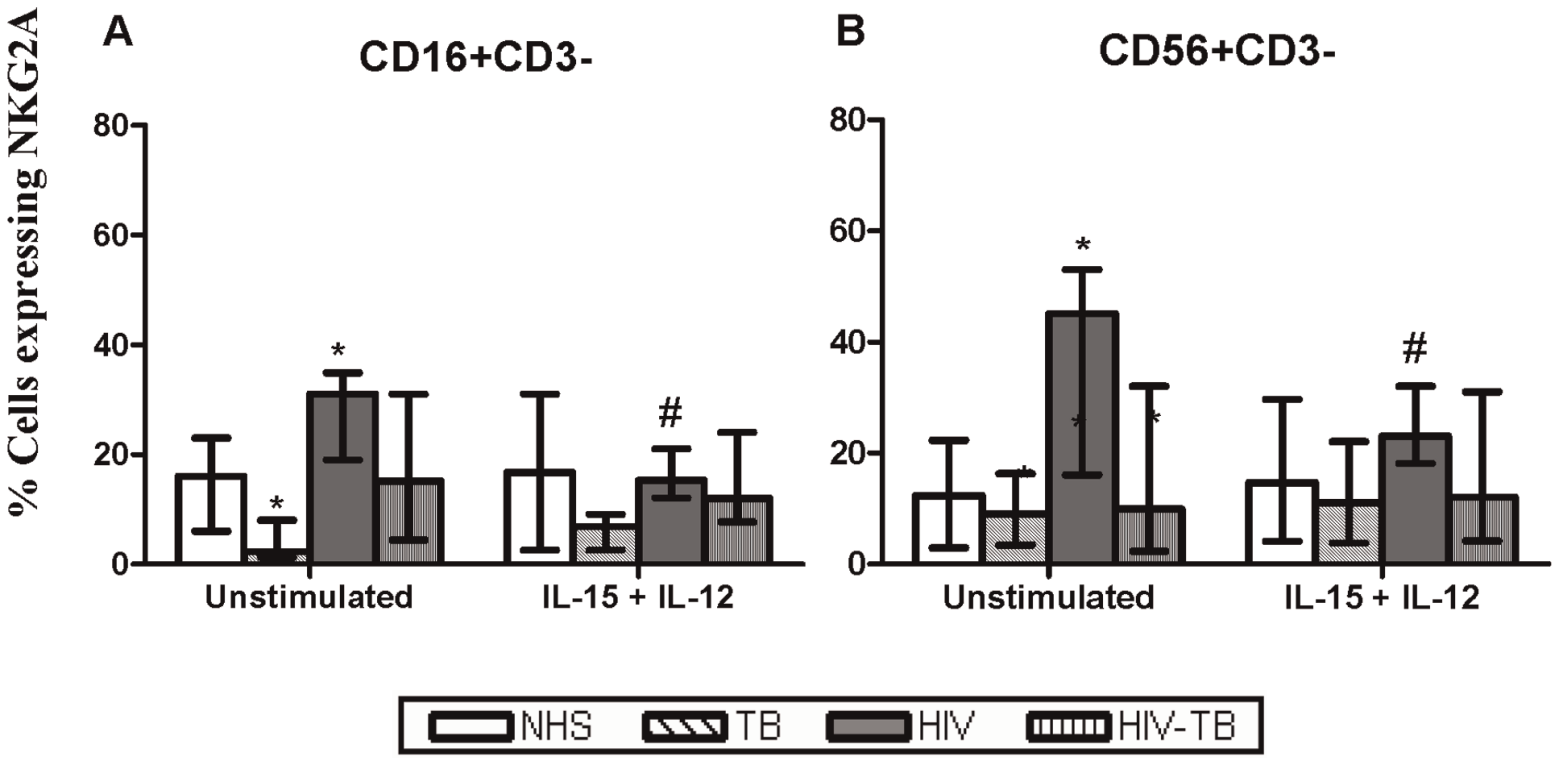
Expression of inhibitory NK receptor NKG2A. NKG2A expression at basal and IL-15+IL-12 stimulation on CD16+CD3− and CD56+CD3− cells from different groups is shown. Data are given as median ± IQR from 15 individuals in each group. Statistical comparisons between NHS and other groups were performed and the significant values (*p*<0.05) are denoted by *. Statistical analysis between basal and IL-15+ IL-12 stimulation were also carried out and the significant values a(*p*<0.05) re showed as #.

The basal expression of NKG2A by NK cell subsets was observed to be reduced in TB compared to NHS. However, NKG2A expression was up-regulated in HIV-infected individuals, which was significant for NK subsets. When the cells were treated with IL-15+ IL-12, there was a significant decline in the expression of this receptor on CD16+CD3− and CD56+CD3− cells in HIV-infected individuals (**Fig. 3**).

### Expression of Activating and Natural Cytotoxicity Receptors (NCRs)

#### NKp46 and NKp30

**Figure 4:**
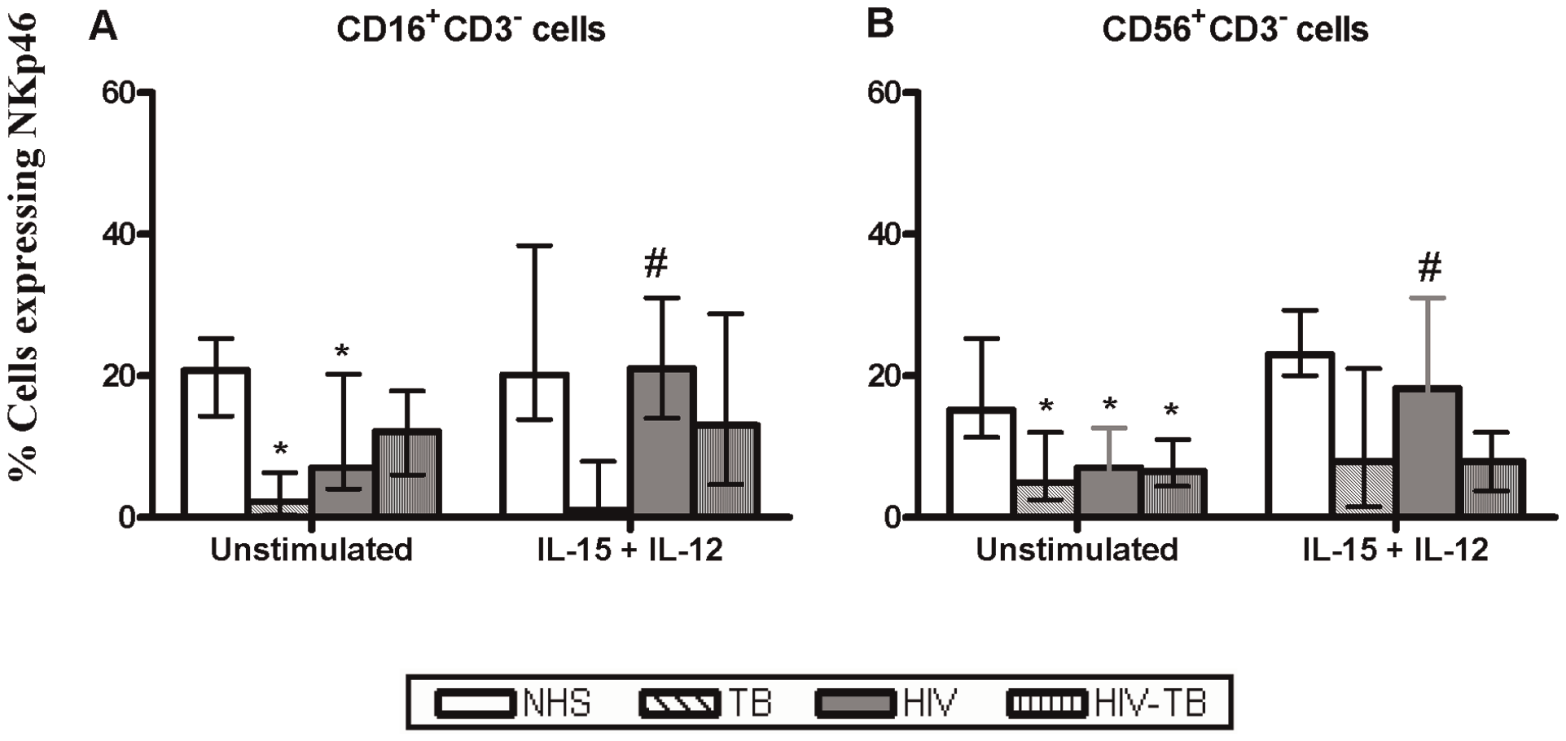
Expression of NKp46. Effect of IL-15+IL-12 on the NKp46 expression from four different groups obtained from 15 individuals. Data for CD16+CD3− and CD56+CD3− subpopulation are presented as median ± IQR. Statistical comparisons between NHS and other groups were performed and the significant values (*p*<0.05) are shown as *. Statistical significance (*p*<0.05) between basal and IL-15+ IL-12 stimulation were represented by #.

In comparison to NHS, the basal frequency of NKp46 on NK subsets was down-regulated (*p*<0.05) in TB, HIV and dually infected individuals. Upon treatment with IL-15+ IL-12, the NK subsets from HIV have shown up-regulation of NKp46 (**Fig. 4**). The expression profile of NKp30 on NK cell subsets after IL-15+ IL-12 stimulation did not differ among groups (**data not shown**).

#### NKp44

The expression of the other NCR, NKp44 by NK cell subsets was found to be lower in TB and HIV-TB, while elevated significantly in HIV-infected individuals, compared to NHS. Stimulation with IL-15+ IL-12 induced elevation of NKp44 expression in NHS, but did not bring about any significant changes in TB, HIV and HIV-TB patients (**data not shown**).

#### NKG2D

**Figure 5:**
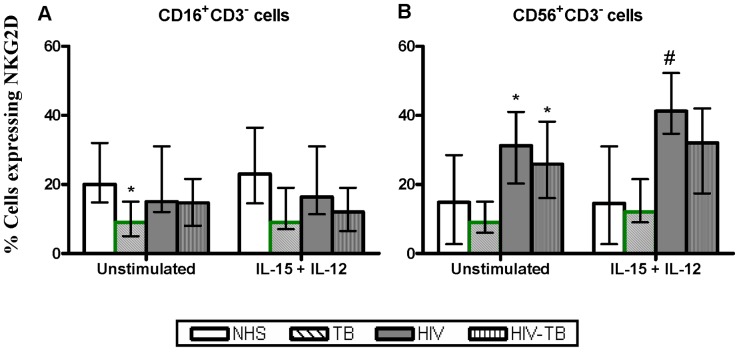
Expression of activatory NK receptor NKG2D. NKG2D expression on CD16+CD3− and CD56+CD3− cells from different groups in the presence/absence of IL-15+IL-12 stimulation is depicted here. Data are given as median ± IQR from 15 individuals each. Statistical comparisons between NHS and other groups were performed and the significant values (*p*<0.05) are denoted by *. Statistical analysis between basal and IL-15+ IL-12 stimulation were also carried out and the significant values a(*p*<0.05) re showed as #.

The number of CD16+CD3− cells expressing NKG2D was found to be lower in TB than NHS. In HIV and HIV-TB, the basal NKG2D expression was higher (*p*<0.05) only for CD56+CD3− cells. Upon stimulation with IL-15+ IL-12, the NKG2D expression among HIV-infected individuals was increased for CD56+CD3− subset but did not affect the CD16+CD3− cells (**Fig. 5**).

### Expression of Coreceptors on NK Cells

#### CD94

The iNKRs coreceptor, CD94 like their dimer NKG2A was found to express proportionately lower by the NK cell subsets in TB and HIV-TB patients. However, CD94 coreceptor was expressed more in HIV-infected individuals. Treatment with IL-15+ IL-12 did not had any effect on NK subsets from the different groups (**data not shown**).

#### NKp80

**Figure 6:**
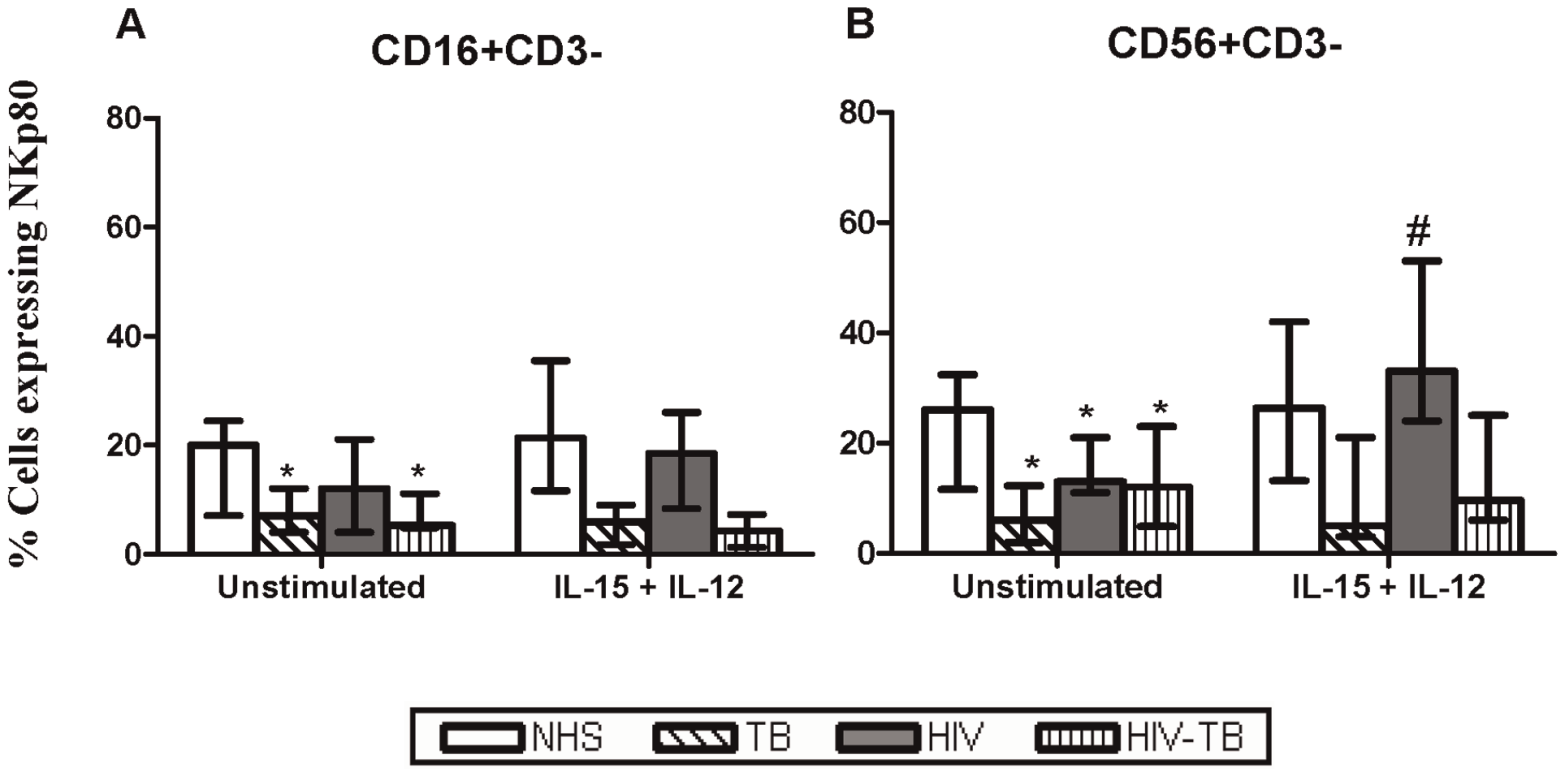
Expression of coreceptor NKp80 on NK cells. The expression of coreceptor NKpp80 at basal and after IL-15+IL-12 stimulation from different groups is shown. Data are given as median ± IQR from 15 individuals in each group. Statistical comparisons between NHS and other groups were performed and the significant values (*p*<0.05) are shown as *. Statistical significance (*p*<0.05) between basal and IL-15+ IL-12 stimulation were represented by #.

The basal expression of NCRs coreceptor, NKp80 on NK cell subsets was reduced in TB, HIV and HIV-TB, compared to NHS. On stimulation, the NKp80 expression from HIV-infected individuals was upregulated by CD56+CD3− cells alone (**Fig. 6**).

#### CD244

The frequency of CD244 expression by NK subsets did not vary between the various groups. Stimulation with IL-15+ IL-12 also had no effect on the CD244 expression in any of the group studied (**data not shown**).

## Discussion

NK cells distinguish between normal healthy cells and abnormal cells by using a sophisticated repertoire of cell surface receptors that control their activation, proliferation, and effector functions. Recent NK cell research has not only revealed insight into the mechanisms of NK cytotoxicity and the regulation via activating and inhibitory receptors, but also pointed out the heterogeneity of NK cells. This cell lineage can be subdivided into several subpopulations according to their functional properties and/or particular phenotypes.

HIV infection is characterized by profound and inappropriate immune activation and progressive immunodeficiency [13], which includes impaired function of NK cells [7] and CD8 cells [14]. The effect of HIV infection on NK cell phenotype and function particularly when co-infected with TB remains to be fully elucidated. In the present study, NK cell subsets from TB, HIV and dually infected individuals were analyzed for the expression of NK cell surface receptors. The CD56 and CD16 cells are examined separately for the different NK receptors and hence the traditional NK subsets are not described. In addition, the effect of *in vitro* IL-15+ IL-12 on the expression of various receptors was investigated from the NK cell subsets of different groups.

The KIR family consists of transmembrane glycoproteins of the Ig superfamily expressed on 1–8% of NK cells, which are involved in recognition of MHC class I molecules on target cells and inhibit cytotoxicity. The CD158a (p58.1) and CD158b (p58.2) molecules regulate NK cell mediated cytolytic activity by interacting with HLA–C alleles [15], while KIRp70 serves as inhibitory receptor for HLA–B molecules [16]. Unlike KIRs that recognize the α1 and α2 domains of human leukocyte antigens with the bound self peptide, the LIRs such as CD85j recognize the nonpolymorphic α3 domain of classical and non-classical HLA molecules. Members of NKG2 family dimerize with CD94 to form a heterodimer that recognize the nonclassical HLA–E molecules [17]. HIV differentially down-regulates MHC class I molecules (HLA–A and B) on target cells to evade killing, but maintains normal expression of HLA–C and E, which are predominant ligands for inhibitory receptors on NK cells [18].

The basal expression of KIR receptors (CD158a, CD158b and KIRp70) on NK subsets was found to be reduced in TB, increased in HIV, while altered in HIV-TB, when compared to NHS. However, the basal expression of other iNKRs (CD85j and NKG2A) from HIV was enhanced (*p*<0.05) in NK subsets. The elevated inhibitory NK receptors in HIV-infected individuals coincides with the previous reports [19], [20], [21]. Up-regulation of iNKRs seems to be a direct effect of HIV infection, since up-regulation of iNKRs was not observed in pulmonary TB patients. In contrast, decreased KIR molecules on CD56+ cells from HIV-infected individuals have also been reported [22], [23].

Stimulation with IL-15+ IL-12 dropped (*p*<0.05) the expression of iNKRs-CD158a, NKG2A and CD85j in HIV-infected individuals which was predominantly observed for CD56+CD3− cells. Reduction in iNKRs during HIV infection might be beneficial to the host as lesser number of ligands is sufficient to activate NK cells. IL-15 has been shown to play a widespread role in the induction of the CD94/NKG2A receptor [24]. Also, transforming growth factor-β (TGF-β), a cytokine with prevalent inhibitory function, has also been shown to induce *in vitro* expression of CD94/NKG2A on CD56+ T-cells [25]. The understanding of cytokine-mediated inhibitory NKR regulation had been so far confined to CD94/NKG2A. HIV-infected individuals in whom viral replication was suppressed by highly active anti-retroviral therapy (HAART), have normal expression of iNKRs on NK cells [20]. Moreover, the NK receptor expression and activity have been shown to normalize in parallel with ART-induced reduction of circulating IL-10 levels [21].

The major NK cell activating receptors are NKG2D and the natural cytotoxicity receptors: NKp30 (NCR1), NKp46 (NCR3) and NKp44 (NCR2) [4]. Fresh NK cells constitutively express NKp30 and NKp46, while the NKp44 receptors are expressed progressively after NK cell activation [26]. To date, the *NKp44* gene has only been identified in humans [27], while genes for other NCR members have also been described in rodents [28], [29]. The cellular ligands of these NCRs have not yet been identified, but studies suggest that they are not expressed on normal tissues, but can be induced under stress and certain pathological conditions, including viral infections [4]. The NKG2D is not related to other highly related NKG2 family members. NKG2D does not form dimers with CD94 and are constitutively expressed on NK cells.

The basal expression of constitutively expressed NCRs (NKp30 and NKp46) on NK cell subsets was lowered (*p*<0.05) in TB, HIV and dually infected individuals as compared to NHS. The expression of NKp44 and NKG2D was found elevated (*p*<0.05) in HIV-infected individuals (mainly CD56+CD3− cells) compared to NHS. The expression of NCRs is markedly decreased among HIV viremic individuals, along with a concomitant decrease in NK cytolytic activity [19], [20], [21], [30], [31]. Though there exist consensus in literature that NKp46 and NKG2D expression on NK cells is reduced in HIV viremia, the basal expression in NHS was reported to vary widely [19], [30], [32], [33], [34]. Possible explanation for this variation include use of peripheral blood lymphocytes vs purified NK cells, analysis within total NK cells vs subsets, geographical variation between cohorts and so on. The percentage of CD56dim cells that express NKp46 and NKp30 and their cytolytic activity decrease with disease progression [30]. On the other hand, concomitant increase of KIR density is also observed on NK cells, thus, setting the basis for an increased inhibitory potential of cytolytic cells [19]. The downregulation of activating receptors and upregulation of KIRs potentiate functional defects in NK-cell-mediated cytotoxicity regardless of the NK cell subpopulation.

The decreased expression of activating and inhibitory NK receptors in TB and HIV-TB might be due to immune deregulation of NK cell function owing to the severe manifestations of TB disease. A direct link between NK cells and *M. tuberculosis*-infected cells have been found to be mediated by the interaction of NKp46- and NKG2D-activating receptors on NK cells with infected monocytes [35]. Patients with recent onset of pulmonary TB have been shown to have low-level expression of both NKp46 and NKp30 [36]. NKp44 has been reported to interact directly to cell-free mycobacteria or other bacteria [37] and to play a role in the recognition of virus-infected cells. Direct interaction of NKp44 with virus-infected cells has also been shown with regard to the envelope protein of flavivirus (WestNile virus, dengue Virus, HCV) [38]. In chronic HCV infection, conflicting evidence with respect to NCRs and NKG2D expression on NK cells have been reported [39], [40].

Stimulation with IL-15+ IL-12 elevated NKp46 (HIV), NKp44 (NHS) and NKG2D (HIV) expression in the CD56+CD3− cells, but did not affect any significant changes in TB and HIV-TB patients. The elevated expression of activating NK receptor might be beneficial to host during HIV infection, as it contributes to more NK cell activation and thereby killing of virus-infected cells. The NK cell expression of NKG2D was increased by culturing with IL-15 or TNF-*α,* but is significantly down-regulated in the presence of TGF-β [41].

CD94 can be expressed on the cell surface as a disulfide-linked homo/hetero dimer with NKG2A or NKG2C. NKG2A has an ITIM in its cytoplasmic domain and CD94/NKG2A heterodimers function as inhibitory receptor [42]. Conversely, CD94/NKG2C heterodimers serve as activating receptors and require association with the DAP12 adapter protein that has ITAM in the cytosplasmic tail, for stable expression on the cell surface and for signaling. Unlike the KIR receptors that are stably maintained once expressed, CD94/NKG2 receptors on NK cells and T cells are modulated by cytokines in the environment. IL-15, TGF-*β* and IL-12 have been shown to induce CD94/NKG2 on human T cells *in vitro* [43].

The present study shows enhanced expression of CD94 on NK subsets in HIV-infected individuals as reported earlier [20] . CD94 *per se* does not bind any MHC ligand, but is essential for the surface expression of NKG2 family of NK receptors [44], [45]. As it may associate with activating (NKG2C) and inhibitory (NKG2A) molecules, it is not clear from the present study whether enhanced expression of CD94 on these cells in HIV-infected individuals is associated with enhanced expression of activating or of inhibitory NKG2 receptors on these cells. Clearly, further work is needed to address this issue.

NKp80 is a type II transmembrane protein with a C-type lectin domain in its extracellular region. The CD244 (2B4) molecule belongs to the Immunoglobulin superfamily of proteins and is structurally related to CD2-like molecules. In humans, CD244 is expressed on NK cells, CD8+ T cells, monocytes and basophils, suggesting a broader role in leukocyte activation [46]. The coreceptors NKp80 and CD244 synergize with NCRs and NKG2D in the NK mediated cytolysis [47].

The NCR coreceptor, NKp80 was found to be expressed in low numbers on CD56+CD3− NK subsets from HIV and HIV-TB compared to NHS. Decreased CD244 expression in HIV-infected, compared to HIV-uninfected individuals was previously observed [20], however, we did not find any significant difference between groups. Stimulation with IL-15+ IL-12 elevated NKp80 expression on CD56+CD3− subset from HIV while stimulation had no effect on CD244 expression. The CD244 expression of NK cells from HIV-infected individuals is believed to vary over time- reduced at 12 months after initiation of HAART, but increased to a level comparable to that of healthy individuals after 36 months [48].

It is interesting in the context of CD56+CD3− NK cells, that it is clear from the data presented that TB patients have different responses to HIV patients and that HIV-TB co-infected patients have similar responses to HIV patients rather than TB patients.

To summarize, IL-15+ IL-12 has an immunomodulatory effect on NK cell subsets from HIV-infected individuals *viz* down-regulation of iNKRs, elevation of activatory receptors NKp46 and NKG2D, and induction of coreceptor NKp80. The effect of IL-15+ IL-12 on NK cell populations of HIV-TB patients is minimal probably due to the influence of tuberculosis. Data from the study revealed that natural killer receptors are differentially expressed and regulated on NK cell subpopulations and are differently affected by HIV. As far as we know, this is the first study to demonstrate the various surface receptors on NK subsets in HIV-infected individuals associated with TB. Further studies on particular NK populations are necessary to identify the main effectors in the interplay of innate and acquired immune functions. Analysis of this interaction is important for the understanding of the NK cell regulation and development of new immune-based interventions in HIV disease.

## References

[1] Biron CA, Nguyen KB, Pien GC, Cousens LP, Salazar-Mather TP (1999). Natural killer cells in antiviral defense: function and regulation by innate cytokines.. Annu Rev Immunol.

[2] Oliva A, Kinter AL, Vaccarezza M, Rubbert A, Catanzaro A (1998). Natural killer cells from human immunodeficiency virus (HIV)-infected individuals are an important source of CC-chemokines and suppress HIV-1 entry and replication in vitro.. J Clin Invest.

[3] Natarajan K, Dimasi N, Wang J, Mariuzza RA, Margulies DH (2002). Structure and function of natural killer cell receptors: multiple molecular solutions to self, nonself discrimination.. Annu Rev Immunol.

[4] Moretta L, Moretta A (2004). Unravelling natural killer cell function: triggering and inhibitory human NK receptors.. Embo J.

[5] Moretta A, Bottino C, Vitale M, Pende D, Biassoni R (1996). Receptors for HLA class-I molecules in human natural killer cells.. Annu Rev Immunol.

[6] Radaev S, Sun PD (2003). Structure and function of natural killer cell surface receptors.. Annu Rev Biophys Biomol Struct.

[7] Ullum H, Gotzsche PC, Victor J, Dickmeiss E, Skinhoj P (1995). Defective natural immunity: an early manifestation of human immunodeficiency virus infection.. J Exp Med.

[8] Ramana Rao PV, Rajasekaran S, Raja A (2008). Augumentation of natural killer activity with exogenous interleukins in patients with HIV and pulmonary tuberculosis coinfection.. AIDS Res Hum Retroviruses.

[9] Fresno M, Kopf M, Rivas L (1997). Cytokines and infectious diseases.. Immunol Today.

[10] Linnemeyer PA (1995). Interleukin-2.. STEP Perspect.

[11] Ramana Rao PV, Rajasekaran S, Raja A (2010). Natural killer cell-mediated cytokine response among HIV-positive south Indians with pulmonary tuberculosis.. J Interferon Cytokine Res.

[12] Ramana Rao PV, Rajasekaran S, Raja A (2010). Natural killer cell-derived cytolytic molecules in HIV-associated pulmonary tuberculosis-Role of exogenous interleukins.. J Clin Immunol: [In Press].

[13] Stevenson M (2003). HIV-1 pathogenesis.. Nat Med.

[14] Lieberman J, Shankar P, Manjunath N, Andersson J (2001). Dressed to kill? A review of why antiviral CD8 T lymphocytes fail to prevent progressive immunodeficiency in HIV-1 infection.. Blood.

[15] Cambiaggi A, Orengo AM, Meazza R, Sforzini S, Tazzari PL (1996). The natural killer-related receptor for HLA-C expressed on T cells from CD3+ lymphoproliferative disease of granular lymphocytes displays either inhibitory or stimulatory function.. Blood.

[16] Moretta L, Biassoni R, Bottino C, Mingari MC, Moretta A (2001). Immunobiology of human NK cells.. Transplant Proc.

[17] Vance RE, Raulet DH (1998). Toward a quantitative analysis of the repertoire of class I MHC-specific inhibitory receptors on natural killer cells.. Curr Top Microbiol Immunol.

[18] Cohen GB, Gandhi RT, Davis DM, Mandelboim O, Chen BK (1999). The selective downregulation of class I major histocompatibility complex proteins by HIV-1 protects HIV-infected cells from NK cells.. Immunity.

[19] Mavilio D, Benjamin J, Daucher M, Lombardo G, Kottilil S (2003). Natural killer cells in HIV-1 infection: dichotomous effects of viremia on inhibitory and activating receptors and their functional correlates.. Proc Natl Acad Sci U S A.

[20] Kottilil S, Shin K, Planta M, McLaughlin M, Hallahan CW (2004). Expression of chemokine and inhibitory receptors on natural killer cells: effect of immune activation and HIV viremia.. J Infect Dis.

[21] Parato KG, Kumar A, Badley AD, Sanchez-Dardon JL, Chambers KA (2002). Normalization of natural killer cell function and phenotype with effective anti-HIV therapy and the role of IL-10.. Aids.

[22] Cauda R, Goletti D, Lucia MB, Tumbarello M, Rumi C (1994). Analysis of natural killer (NK) cell subsets defined by the expression of two novel surface antigens (EB6 and GL183) in AIDS and AIDS-related conditions.. Clin Immunol Immunopathol.

[23] Ahmad R, Sindhu ST, Tran P, Toma E, Morisset R (2001). Modulation of expression of the MHC class I-binding natural killer cell receptors, and NK activity in relation to viral load in HIV-infected/AIDS patients.. J Med Virol.

[24] Mingari MC, Vitale C, Cantoni C, Bellomo R, Ponte M (1997). Interleukin-15-induced maturation of human natural killer cells from early thymic precursors: selective expression of CD94/NKG2-A as the only HLA class I-specific inhibitory receptor.. Eur J Immunol.

[25] Bertone S, Schiavetti F, Bellomo R, Vitale C, Ponte M (1999). Transforming growth factor-beta-induced expression of CD94/NKG2A inhibitory receptors in human T lymphocytes.. Eur J Immunol.

[26] Biassoni R, Cantoni C, Pende D, Sivori S, Parolini S (2001). Human natural killer cell receptors and co-receptors.. Immunol Rev.

[27] Cantoni C, Bottino C, Vitale M, Pessino A, Augugliaro R (1999). NKp44, a triggering receptor involved in tumor cell lysis by activated human natural killer cells, is a novel member of the immunoglobulin superfamily.. J Exp Med.

[28] Biassoni R, Pessino A, Bottino C, Pende D, Moretta L (1999). The murine homologue of the human NKp46, a triggering receptor involved in the induction of natural cytotoxicity.. Eur J Immunol.

[29] Pende D, Parolini S, Pessino A, Sivori S, Augugliaro R (1999). Identification and molecular characterization of NKp30, a novel triggering receptor involved in natural cytotoxicity mediated by human natural killer cells.. J Exp Med.

[30] Mantegani P, Tambussi G, Galli L, Din CT, Lazzarin A (2010). Perturbation of the natural killer cell compartment during primary human immunodeficiency virus 1 infection primarily involving the CD56 bright subset.. Immunology.

[31] De Maria A, Fogli M, Costa P, Murdaca G, Puppo F (2003). The impaired NK cell cytolytic function in viremic HIV-1 infection is associated with a reduced surface expression of natural cytotoxicity receptors (NKp46, NKp30 and NKp44).. Eur J Immunol.

[32] Andre P, Castriconi R, Espeli M, Anfossi N, Juarez T (2004). Comparative analysis of human NK cell activation induced by NKG2D and natural cytotoxicity receptors.. Eur J Immunol.

[33] Bozzano F, Nasi M, Bertoncelli L, Nemes E, Prati F (2011). NK-cell phenotype at interruption underlies widely divergent duration of CD4+-guided antiretroviral treatment interruption.. Int Immunol.

[34] Fu GF, Hao S, Zhao JL, Xu XQ, Guo HX (2009). Changes in NK cell counts and receptor expressions and emergence of CD3(dim)/CD56+ cells in HIV-1 infected patients in China.. Viral Immunol.

[35] Vankayalapati R, Wizel B, Weis SE, Safi H, Lakey DL (2002). The NKp46 receptor contributes to NK cell lysis of mononuclear phagocytes infected with an intracellular bacterium.. J Immunol.

[36] Bozzano F, Costa P, Passalacqua G, Dodi F, Ravera S (2009). Functionally relevant decreases in activatory receptor expression on NK cells are associated with pulmonary tuberculosis in vivo and persist after successful treatment.. Int Immunol.

[37] Esin S, Batoni G, Counoupas C, Stringaro A, Brancatisano FL (2008). Direct binding of human NK cell natural cytotoxicity receptor NKp44 to the surfaces of mycobacteria and other bacteria.. Infect Immun.

[38] Hershkovitz O, Rosental B, Rosenberg LA, Navarro-Sanchez ME, Jivov S (2009). NKp44 receptor mediates interaction of the envelope glycoproteins from the West Nile and dengue viruses with NK cells.. J Immunol.

[39] Ahlenstiel G, Titerence RH, Koh C, Edlich B, Feld JJ, et al. Natural killer cells are polarized toward cytotoxicity in chronic hepatitis C in an interferon-alfa-dependent manner.. Gastroenterology.

[40] De Maria A, Fogli M, Mazza S, Basso M, Picciotto A (2007). Increased natural cytotoxicity receptor expression and relevant IL-10 production in NK cells from chronically infected viremic HCV patients.. Eur J Immunol.

[41] Castriconi R, Cantoni C, Della Chiesa M, Vitale M, Marcenaro E (2003). Transforming growth factor beta 1 inhibits expression of NKp30 and NKG2D receptors: consequences for the NK-mediated killing of dendritic cells.. Proc Natl Acad Sci U S A.

[42] Houchins JP, Lanier LL, Niemi EC, Phillips JH, Ryan JC (1997). Natural killer cell cytolytic activity is inhibited by NKG2-A and activated by NKG2-C.. J Immunol.

[43] De Maria A, Moretta L (2000). HLA-class I-specific inhibitory receptors in HIV-1 infection.. Hum Immunol.

[44] Lanier LL, Phillips JH (1996). Inhibitory MHC class I receptors on NK cells and T cells.. Immunol Today.

[45] Yokoyama WM (1998). HLA class I specificity for natural killer cell receptor CD94/NKG2A: two for one in more ways than one.. Proc Natl Acad Sci U S A.

[46] Mason D, Andre P, Bensussan A, Buckley C, Civin C (2002). CD antigens 2002.. Blood.

[47] Moretta A, Bottino C, Vitale M, Pende D, Cantoni C (2001). Activating receptors and coreceptors involved in human natural killer cell-mediated cytolysis.. Annu Rev Immunol.

[48] Ostrowski SR, Ullum H, Pedersen BK, Gerstoft J, Katzenstein TL (2005). 2B4 expression on natural killer cells increases in HIV-1 infected patients followed prospectively during highly active antiretroviral therapy.. Clin Exp Immunol.

